# A Literature Review of the Future of Oral Medicine and Radiology, Oral Pathology, and Oral Surgery in the Hands of Technology

**DOI:** 10.7759/cureus.45804

**Published:** 2023-09-23

**Authors:** Ishita Singhal, Geetpriya Kaur, Dirk Neefs, Aparna Pathak

**Affiliations:** 1 Oral Pathology and Microbiology and Forensic Odontology, Shree Guru Gobind Singh Tricentenary (SGT) University, Gurugram, IND; 2 Oral Pathology and Microbiology, Paradise Diagnostics, New Delhi, IND; 3 Dentistry, Dierick Dental Care, Antwerp, BEL; 4 Oral Pathology, Paradise Diagnostics, New Delhi, IND

**Keywords:** oral and maxillofacial pathology, oral and maxillofacial surgery, oral medicine and radiology, extended reality, mixed reality, artificial intelligence (ai), virtual reality (vr), augmented reality (ar), ai and robotics in healthcare

## Abstract

In the realm of dentistry, a myriad of technological advancements, including teledentistry, virtual reality (VR), artificial intelligence (AI), and three-dimensional printing, have been extensively embraced and rigorously evaluated, consistently demonstrating their remarkable effectiveness. These innovations have ushered in a transformative era in dentistry, impacting every facet of the field. They encompass activities ranging from the diagnosis and exploration of oral health conditions to the formulation of treatment plans, execution of surgical procedures, fabrication of prosthetics, and even assistance in patient distraction, prognosis, and disease prevention. Despite the significant strides already taken, the relentless pursuit of new horizons fueled by human curiosity remains unabated. The future landscape of dentistry holds the promise of sweeping changes, notably characterized by enhanced accessibility to dental care and reduced treatment durations. In this comprehensive review article, we delve into the pivotal roles played by AI, VR, augmented reality, mixed reality, and extended reality within the realm of dentistry, with a particular emphasis on their applications in oral medicine, oral radiology, oral surgery, and oral pathology. These technologies represent just a fraction of the technological arsenal currently harnessed in the field of dentistry. A thorough comprehension of their advantages and limitations is imperative for informed decision-making in their utilization.

## Introduction and background

The world has witnessed remarkable evolution across numerous domains, with technology standing as a pivotal driver of this transformation. In the realm of healthcare, technology has ushered in a wave of innovations that encompass artificial intelligence (AI), virtual reality (VR), augmented reality (AR), mixed reality (MR), extended reality (XR), and robotic applications, all aimed at enhancing patient care. While concerns about technology encroaching on human roles in healthcare persist, there is a prevailing belief that these advancements can empower healthcare professionals to deliver superior patient care. This empowerment comes in the form of augmented decision-making capabilities, improved long-term patient tracking, expedited image analysis, and exceptional predictive capabilities. Notably, AI and machine learning (ML) are seen as complementary tools that enhance human expertise rather than replace it. As with any innovation, these technologies come with their own set of advantages and challenges. In this comprehensive review article, we delve into the multifaceted roles of AI, VR, AR, MR, and XR within the field of dentistry. Our focus will be on their applications in oral medicine, oral radiology, oral surgery, and oral pathology, providing insights into how these technologies are reshaping and optimizing dental practice for both professionals and patients.

AI encompasses the theory and development of computer systems capable of performing tasks typically associated with human intelligence, such as visual perception, speech recognition, decision-making, and language translation [1]. In the realm of healthcare, AI finds practical applications in advanced online search engines, recommendation systems, and creative tools [2]. One subset of AI, known as ML, focuses on the development of computer algorithms and models that enable systems to learn from and make predictions or decisions based on data, without being explicitly programmed to perform specific tasks. In essence, ML allows computers to recognize patterns, make sense of data, and improve their performance or behavior over time through experience [3]. In the context of dentistry, AI has been harnessed to automatically analyze dental X-rays, yielding crucial insights such as X-ray type, potential tooth impact, precise degree of bone loss through color overlays, cavity location, and more [4]. Deep learning (DL), a specialized branch of ML characterized by multi-layered computational networks, has emerged as a game-changer, particularly in medical and dental image processing [5,6]. The promise of ML and DL in dentistry extends to enhanced diagnostic accuracy and treatment planning. For instance, a noteworthy development at the University of California involved the creation of an AI algorithm with a remarkable 94% accuracy rate in detecting periodontitis. This algorithm exhibited diagnostic accuracies of 73% for distinguishing normal from diseased cases and 59% for classifying the severity levels of bone loss. Further optimization of the periodontal dataset holds the potential to transform this computer-aided detection system into an efficient tool for periodontal disease detection and staging [7]. In addition to periodontitis, DL algorithms have also proven adept at accurately identifying dental caries in X-rays. These technologies, characterized by their objectivity and reduced bias, hold the promise of revolutionizing dentistry by standardizing and improving the diagnostic process [6].

VR is a computer-generated simulation of a hypothetical, immersive, three-dimensional (3D) world or picture that may be interacted with using certain technologies [8]. VR has been utilized in medicine to great effect as a distraction tool during operations as well as an acclimatization technique to prepare for an experience or procedure, as stated in systematic reviews and randomized control experiments [9]. Although it has not yet gained widespread acceptance in dentistry, it may potentially play a part in exposure-based acclimation to dental events. In comparison to no intervention, three trials employing VR in a dental context found reduced pain and anxiety. The perioperative phase served as the setting for all three of these studies [9]. VR may be employed to eradicate dental phobia in pediatric and geriatric patients and further enhance patient education. Before performing procedures on actual patients, dentists, and dental students can practice and test them on mannequins using VR technology by utilizing 3D models of teeth or a human head. Additionally, VR may be used to teach new dentists and make sure seasoned dentists maintain their skill sets [10].

AR is a technique that superimposes digital data over the physical environment. Incorporating computer-generated sensory input like audio, video, graphics, or GPS data improves the user's impression of reality [11]. By immediately displaying healthcare data on the patient and merging the physical and digital worlds, AR primarily seeks to improve clinical practice. By adopting interactive approaches, AR and VR technology can help dentists explain various dental operations to their patients, establish a diagnosis, create a treatment plan, and clearly illustrate predicted results by using 3D models of their patient's teeth, gums, and oral cavities [12].

VR and AR are both combined in MR. It makes it feasible to embed features in a real setting by enabling digital things to interact with the physical world. MR equipment can be utilized in dentistry for surgical planning and training. It could offer a fresh idea for people getting dental treatment when it comes to giving consent. The Microsoft HoloLens is an MR device that can display information and potentially create a virtual environment utilizing a real-time, 3D platform employing several sensors and holographic processing. The HoloLens technology may be utilized as an essential tool for dentistry education and surgery planning, given how quickly technology is developing and how popular virtual learning is becoming [13,14].

XR is an umbrella term that encompasses all types of technologies that enhance our senses, including VR, AR, and MR [15]. Additionally, these technologies have been applied in several industries, including entertainment, education, and health care. It is a notion that covers both tangible and fictitious hybrid worlds, as well as human-machine interactions produced through wearables and computer technologies. Implantology and orthognathic procedures are the two dental uses of XR that occur most frequently. The development of reality gadgets makes it possible for users to mix and include both medical data and graphical information. Dental implant virtual planning, which transposes 3D virtual planning into the surgical field, has increased the precision of dental implants being inserted using either static guiding or dynamic navigation. Dental static-guided devices may not offer as many benefits in dental implantology as computer-assisted surgery with dynamic navigation. These kinds of technologies overlay computed cone-beam tomography (CBCT) depth, angle, and drill position on the pictures, assisting dentists in performing minimally invasive procedures and avoiding damage to important structures. Because computer-aided navigation increases treatment precision while lowering operational hazards, the adoption of such technology is also beneficial in oral and maxillofacial surgery. Users may occasionally wear a head-mounted display or a glove that stimulates their visual, auditory, and tactile senses, as well as their sense of touch, to create an immersive virtual experience.

Currently, mock-ups, video analysis, and 3D face conceptions have all been employed to help with the technique of reconstructing smiles during dental rehabilitation. The development of new technology improves this program and cuts down on the amount of time and chance of mistakes involved in knowledge exchange between patients, physicians, and laboratories. Increased realism grin programs locate the smile in the photograph and replace it with a different smile for the greatest fit [15].

Technology has indeed revolutionized the field of dentistry, making it more comfortable, efficient, and effective for patients. Other technical developments that have influenced modern dentistry include laser dentistry, CAD/CAM, 3D printing, and regenerative dentistry. In addition to these developments in dentistry, these technologies are also having a big impact on how dental care is provided.

## Review

History

**Figure 1:**
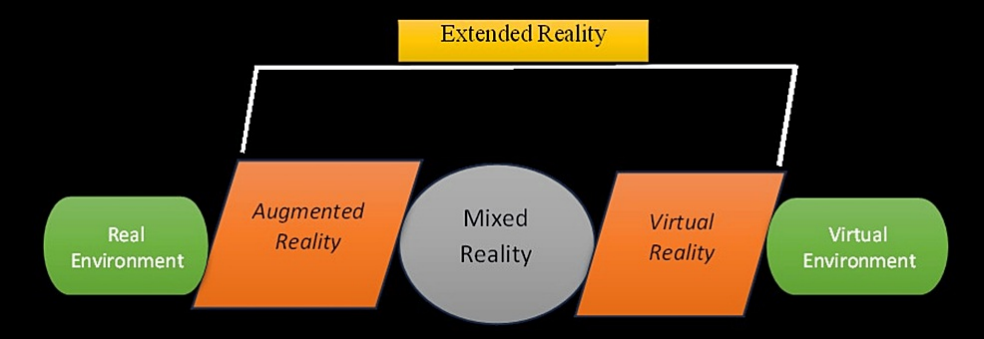
Diagrammatic representation of reality-virtuality continuum. Image Credits: Geetpriya Kaur

In the current decade, we are gradually entering the realm of the fourth dimension, where experiences previously unattainable in the physical world become accessible. The history of AI can be traced back to 1956, while the origins of VR reach back to 1960 [16]. Notably, in 1962, Morton Heiling pioneered the Sensorama technology, a multisensory stimulator that incorporated color and stereo-prerecorded films, augmented by binaural scents, sound, wind, and vibration backgrounds. However, Sensorama's interactivity was limited [17]. In 1965, Ivan Sutherland demonstrated "The Ultimate Display" technology, based on the concept of constructing an artificial world that integrated interactive graphics, force feedback, olfactory, gustatory, and auditory elements [18]. Furthermore, in 1968, Sutherland introduced the first head-mounted display (HMD) system with a three-dimensional head-tracking method, aptly named "The Sword of Damocles" [19]. The year 1971 marked the development of GROPE, the first prototype of a force-feedback system at the University of North Carolina (UNC). GROPE combined haptic display and visual models [16]. In 1975, Myron Krueger established an artificial reality laboratory known as the Videoplace, creating a "conceptual environment that did not exist." This system displayed user silhouettes captured by cameras on a large screen, enabling user interaction [20]. In 1982, Thomas Furness pioneered the Visually Coupled Airborne System Simulator (VCASS), an advanced flight simulator demonstrated at the US Air Force’s Armstrong Medical Research Laboratories. Fighter pilots utilized an HMD that integrated the outside view with graphics displaying precise flight path information [16]. In 1984, NASA Ames developed the Virtual Visual Environment Display (VIVED), featuring a stereoscopic monochrome HMD [21]. The VPL company made significant strides in commercial VR technology, introducing the iconic Data Glove technology in 1985 and the marketable Eyephone HMD in 1988 [16]. In 1989, Fake Space Labs introduced the BOOM technology, a compact device composed of two CRT monitors viewable through eye holes [16]. The latter part of the 1980s witnessed the creation of numerous VR devices, including optical trackers, HMDs, and the Pixel-Plane graphics engine at UNC. The architectural walkthrough was a notable application of these technologies. In 1992, the CAVE Automatic Virtual Environment emerged, amalgamating VR and scientific visualization systems. Users wore LCD shutter glasses, and stereoscopic images were projected onto the room's walls, offering high-resolution images and a wider field of view compared to HMDs [21]. In 1994, Milgram and Kishino introduced the concept of the VR continuum, encompassing five systems: AI, AR, VR, MR, and XR (Figure 1) [16]. AR technology, within this continuum, enhances rather than replaces the real world. AR employs see-through HMDs to overlay virtual three-dimensional objects onto the real environment. AR holds substantial potential for enhancing human perception and facilitating complex tasks, making it a focal point of various research endeavors [22].

Oral medicine

Virtual Reality and Augmented Reality in Oral Medicine

Conventionally, oral cavity examinations and clinical diagnostic investigations of oral lesions were either explained orally or with visual presentations. The oral medicine residents are expected to take a proper clinical history of the patient with a thorough oral cavity examination. A case history can be described as a planned professional conversation that enables the patient to communicate his or her symptoms and past personal, dental, and medical histories [23]. A 3D-augmented clinical history format can be created to record the chief complaint, medical and dental histories, as well as previous investigations. Hence, utilizing this platform will greatly help in making a provisional diagnosis of the patient and explaining the patient with the help of images.

The most common types of oral lesions encountered by an oral medicine resident are red and white lesions, vesiculobullous lesions, and ulcerative lesions. The conventional chairside diagnostic techniques include vital staining, exfoliative cytology, and optical imaging. A demonstration of these methods can be explained through a 3D-augmented platform. Haptic-based VR training stimulators can be used by oral medicine residents for the practice of these traditional techniques. Additionally, VR can also assist the dentist in ruling out false-positive and false-negative results of several vital staining procedures, including toluidine blue, methylene blue, and Lugol’s iodine.

Some oral lesions are treated with medication, while other oral lesions are recommended for biopsy. An oral premalignant condition such as oral submucous fibrosis is generally treated with hyaluronidase injections. The placement of injections and dosage can be demonstrated with the help of VR training stimulators. In the case of white lesions, the application of medications or oral intake of medicines can be explained with stimulators [23].

**Figure 2:**
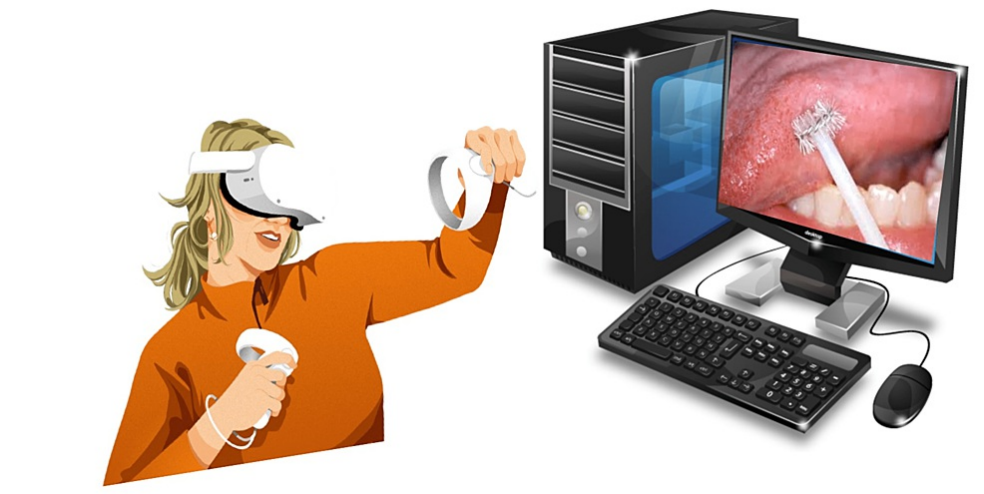
An oral medicine resident using a virtual reality device to grasp an exfoliative smear procedure with a cytobrush. Image Credits: Geetpriya Kaur and Ishita Singhal

Incisional and punch biopsy techniques are generally preferred to evaluate potentially malignant oral disorders and oral squamous cell carcinoma. Excisional biopsy procedures are performed in cases of exophytic growth, pyogenic granuloma, and mucocele. The AR stimulators can be used for explaining and practicing biopsy procedures to oral medicine residents. Moreover, the tactile feedback mechanisms can be deeply studied to enhance biopsy procedures (Figure 2).

Artificial Intelligence in Oral Medicine

AI is nowadays a popular diagnostic modality that is being used for precise image analysis by making use of various body systems. The various AI techniques that are currently being utilized are artificial neural networks (ANNs) and genetic algorithms. In the recent decade, ANNs have been used to elucidate the findings of various investigative modalities like USG, dental radiographs, CBCT, computed tomography (CT) scans, and magnetic resonance imaging (MRI). Moreover, by using ANNs, we can manage precise generalization of settings by optimizing the goodness of fit between the input data (text or image fed into the algorithm) and output data (working classification). Additionally, ML algorithms can also provide accurate clinical findings by analyzing hospital medical records that have been hand-labeled [24].

Additionally, AI can be used as an adjunct for diagnosing oral lesions as well as planning efficacious treatment based on clinical findings. For example, AI algorithms can help in the classification of various suspicious lesions that might be undergoing malignant changes. Thus, in future research, AI can also be judiciously used for screening larger populations for genetic predisposition to oral cancer. Moreover, AI can also provide supportive diagnostic acumen along with other chief diagnostic techniques like CT, MRI, and CBCT to determine certain deviations from the normal anatomical arrangement that might have been missed by the human eye [24].

Oral radiology

Oral radiology is a specialized field of dentistry that employs various imaging methods to diagnose and treat oral diseases. Its primary role is to identify pathologies such as cysts, tumors, and infections in the oral cavity. In oral radiology, a range of imaging techniques are utilized, including radiographs, CBCT, CT scans, MRI, positron emission tomography (PET) scans, and ultrasound (USG). Radiographs are commonly employed for the detection of dental caries, periodontal disease, cysts, benign and malignant tumors, and other dental abnormalities. CT scans are particularly useful in assessing bone loss, fractures, and tumors, while MRI is effective in detecting soft tissue abnormalities such as cysts and tumors. Ultrasound is primarily utilized to evaluate salivary gland irregularities. This branch of dentistry plays a critical role in effectively diagnosing and treating oral ailments [25].

Artificial Intelligence in Oral Radiology

**Figure 3:**
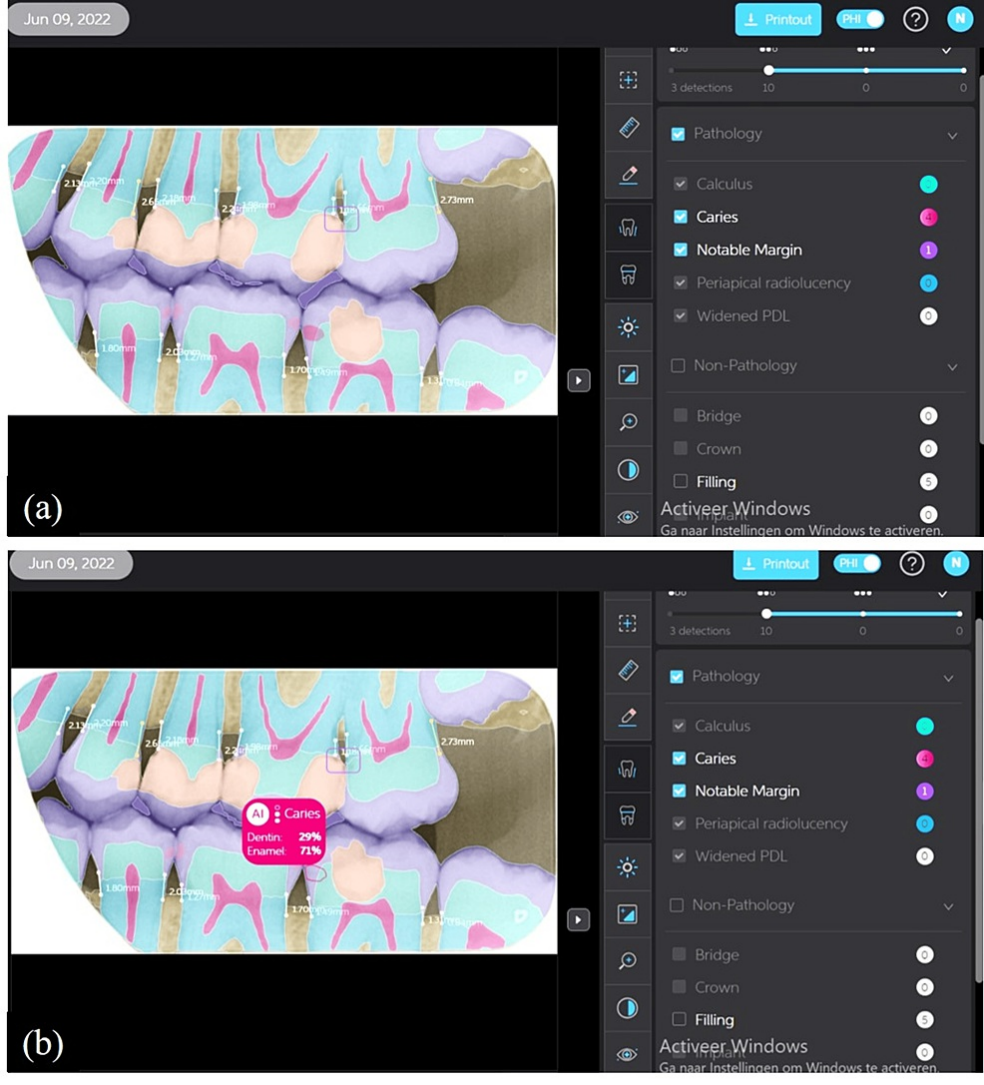
Role of artificial intelligence in the annotation of dental X-rays. (a) Segmentation of the different tooth structures and (b) details on the penetration level of the tooth decay. Image Credits: Dirk Neefs, DD-Care, Belgium

In the field of oral and maxillofacial radiology, AI applications hold considerable promise. CNNs, which can perform image categorization, detection, segmentation, registration, creation, and refinement, have been primarily employed in recent research on AI in oral and maxillofacial radiology. In this area, AI algorithms have been created for image analysis, forensic dentistry, radiographic diagnostics, and picture quality enhancement. Dental radiology is steadily integrating AI, with a focus on diagnostic records in CBCT and digital 3D images. To develop AI for quick diagnosis and improved treatment planning, a lot of data may be gathered and calculated. To get good results, a ton of data is required, and oral radiologists must be involved in this labor-intensive process of creating accurate and consistent data sets. There are several issues that need to be resolved before AI is extensively used in current clinical practice, including the need to build up enormous amounts of finely labeled open data sets, comprehend AI's judgment standards, and identify potential AI-based threats to DICOM. AI will advance further in the future and is anticipated to play a significant role in the creation of automatic diagnosis systems, the establishment of treatment plans, and the manufacture of treatment instruments if answers to these issues are offered with the growth of AI. As specialists who are well-versed in the properties of radiographic pictures, oral radiologists will be especially crucial in the development of AI applications in this area (Figure 3) [25].

Virtual Reality and Augmented Reality in Oral Radiology

**Figure 4:**
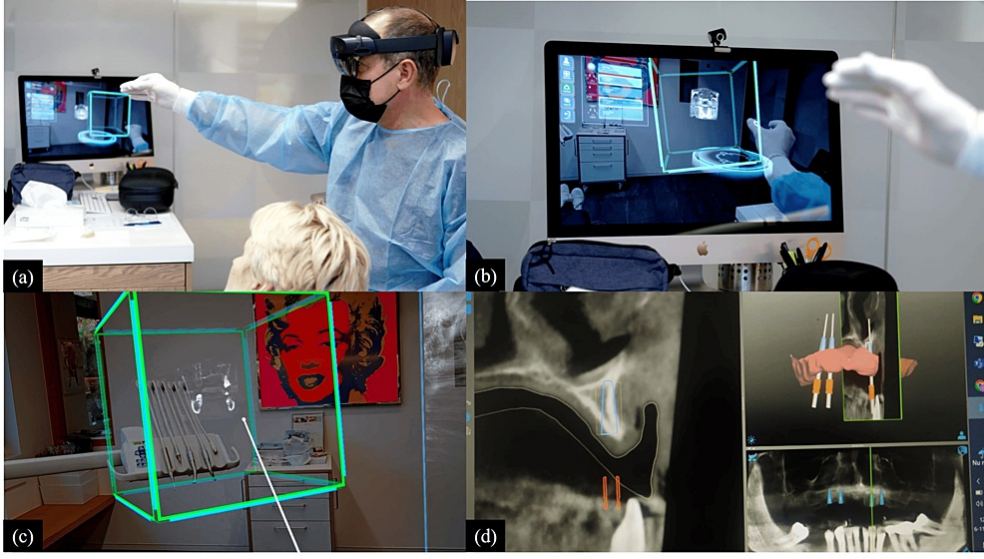
(a), (b), and (c) Application of augmented reality in oral radiology and mixed reality in oral surgery, the surgeon visualizes the medical images as holograms. (d) The digital preparation of dental implant planning. This planning is used to fabricate a drill guide. Image Credits: Dirk Neefs, DD-Care, Belgium

The use of virtual and augmented reality technology is a cutting-edge method of communication that has the potential to enhance radiology education, improve communication with coworkers, refer physicians and patients, and support interventional radiology operations. Currently, AR and VR technologies only allow the user to view content; they do not allow them to interact with the environment or receive tactile input from it. New technologies enable interaction and manipulation of the environment by people. Anatomical holograms may be "tagged" to manipulable actual items using low-cost, commercially accessible equipment like the MERGE Cube (Merge VR, San Antonio, Texas). Although VR and AR have a lot of potential for radiography, they currently have certain drawbacks, such as ergonomic issues from extended usage, relatively high adoption and use costs, and a lack of content. For instance, it has been noted that continuous usage of HMDs might result in neck discomfort, nausea, and disorientation from prolonged delay (Figure 4) [26].

Oral surgery

Virtual Reality, Augmented Reality, and Mixed Reality in Oral Surgery

An oral and maxillofacial surgeon should have precise knowledge of anatomical structures and their normal physiological movements [27]. For better surgical results, AR-VR technology can provide visual access and detailed knowledge of important anatomical structures, muscles, and joint movements of the oral cavity to oral surgery residents. Based on clinical and radiological investigations, VR devices can be used to plan customized patient treatment. The Holomedicine® Association is a global network of individual experts from medicine, science, technology, and policy. They work to build new methods for delivering mixed reality technologies in medicine and surgery, ensuring they have maximum clinical impact.

Oral surgeons can be taught the administration of dental anesthesia with the help of the AR-VR platform. The main reasons for anesthesia failures are anatomical, pathological, physiological, or inappropriate techniques [28]. Additionally, anatomical and inappropriate insertion techniques can be further improved by imparting a deep knowledge of intraoral and extraoral anatomies [27]. This technology, if integrated with a feedback mechanism, can be greatly beneficial in handling larger groups of patients in a short period. A study determined the dentist feedback of haptic-based VR anesthesia injection training stimulators on two different virtual models. Although the results were satisfactory, the enhancement of tactile feedback was the main concern [28]. Another study compared inferior alveolar nerve block (IANB) teaching methods in an AR stimulator-based experimental group and a conventional technique-based control group. However, the researchers did not discuss the limitations of the feedback system [29].

In the recent decade, AR techniques have been used to assist in several oral and maxillofacial surgeries, such as orthognathic surgery, osteotomies, prosthetic surgeries, cancer surgery, temporomandibular joint analysis, excisional biopsy procedures, and dental implants [30]. An image-guided surgery system was developed using AR technology. A computer-assisted system consisted of a gadget that could monitor the instrument, whose position and direction were depicted in virtual space using a picture registration process [31]. Computer design methods were used to connect the instrument to the surgical field. Generally, oral surgeons use a pointer to connect preoperative patient images and the surgical treatment plan. Moreover, AR-based technology was developed to project pictures directly onto the surgical site. This technique uses mononuclear projection in the working microscope and head-up displays. The projections were further built on the semi-clear screens and placed between the working screen and the oral surgeon, or they were constructed on the binocular optics of a following surgical microscope [32].

In the latest study, oral surgeons used HMD to understand the superimposition of bone segments or soft tissues. This technique helped to accomplish a smoother surgical performance [33]. In another study, Oculus Rift and Leap Motion equipment were used by residents to interact with the equipment and understand its operations [34]. The Leap Motion system integrates a multi-sensory learning experience by using a particular application and zooming in on some treatment techniques within possible oral surgical procedures. This VR technology incorporated a 360-degree operating room, spherical videos, and computer-generated three-dimensional operating room models [27]. Still, further studies are required to understand the haptic force feedback and its association with the three-dimensional instrument models.

Some researchers utilized the VR platform to perform virtually simulated orthognathic surgeries and later carried out these surgical interventions on patients. The major benefit of this technology was that oral surgeons could predict patients’ aesthetic and surgical progress [35]. The AR framework was used for tracing points, lines, and planes that could be moved from the stereolithographic skull model on the facial skeleton during osteotomy and splint procedures [31]. The VR devices were also used to study the placement of dental implants. Proper surgical navigation can be used by dental surgeons to place the implants at specific locations with sufficient bone thickness, thereby preventing implant failure [18]. Thus, VR technology has been judiciously incorporated to provide proper treatment planning and determine a precise location [36].

**Figure 5:**
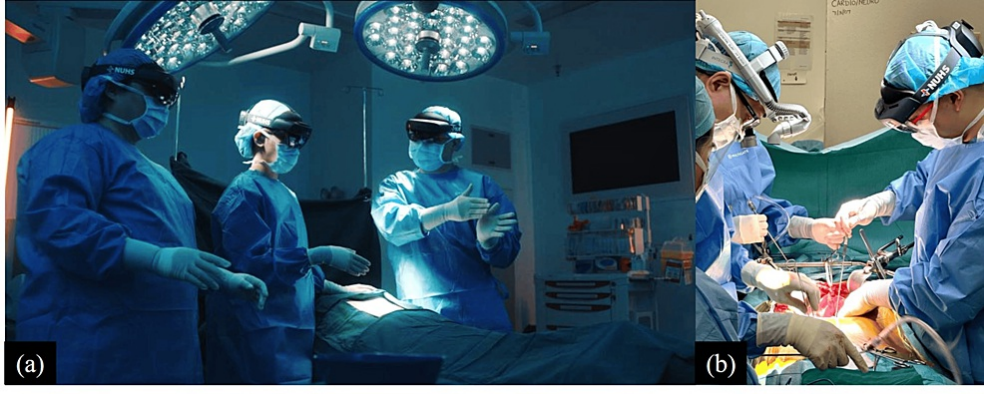
Oral surgery residents using mixed reality devices (a) to precisely study patients’ facial anatomy pre-operatively, and (b) while operating on the patient. Image Credits: Yujia Gao (NUHS, Singapore) provided their original images for publication.

Siepel et al. examined the application of a low-cost stereoscopic display system and six degrees of freedom in implant placement and compared it with the three degrees of freedom in the virtual world. During the follow-up research, the treatment planning was enhanced so that the dentist was provided with six degrees of freedom using CT images at the voxel level in real time [37]. The latest studies demonstrated VR systems that integrated CT images of the jawbone along with haptic force feedback technology to train beginners by simulating the sounds and vibrations of bone drilling and contra-angled handpieces [38,39]. In oncology cases, the oral and maxillofacial consultant can use VR technology to physically draw the tumor borders with the help of programming apparatuses onto the processed tomographic informational index (Figure 5) [40].

Artificial Intelligence in Oral Surgery

In many recent studies, machine learning algorithms have been utilized for faster and more reproducible interpretation of several bone and skin landmarks that are necessary for a complete 3D analysis of facial structures. Thus, this technique has more potential than other computational techniques [41].

For managing orofacial deformities, the choice of surgery is important, along with the expertise of the orthodontist and the surgeon. Hence, training algorithms on cephalometric values as well as interpreted images can help in providing treatment support tools that can easily predict the requirement for surgical interventions during orthodontic treatment. Moreover, these AI-based tools can lend a helping hand to the assisting practitioner to either verify or revise his treatment plans accordingly to minimize orthodontic camouflage with adverse aesthetic and functional results [41].

The extraction of impacted third molars is a routine procedure performed by oral surgeons and general dentists. The use of AI-based tools can help optimize the various stages of diagnosis and treatment planning. Moreover, taking the support of a predictive AI-based model based on the eruption potential of the third molars by means of mechanized calculations of their angulation on panoramic radiographs can judiciously help in making crucial decisions pertaining to tooth extraction, which might turn out to be debatable in a few cases [40,41].

Oral pathology

Artificial Intelligence, Virtual Reality, and Augmented Reality in Oral Pathology

An oral pathologist evaluates the relevant stained tissue slides under a microscope and provides the patient with a histopathology report. The final diagnosis of oral lesions greatly depends on accurate clinical and radiological patient details. Furthermore, oral surgeons can further provide the required treatment to patients based on their histopathology reports.

In recent decades, light microscopy has been replaced by digital scanning techniques. However, hematoxylin and eosin (H&E) staining is still used as the standard method. It is predicted that an oral pathologist will soon be able to directly examine the oral tissues without any tissue processing steps. Thus, 3D AR or MR technology could be precisely applied in this research area for better patient outcomes. In the future, the oral tissues could be directly viewed in the patient’s mouth by utilizing in vivo microscopy to ultimately connect relevant cellular features to matching radiological images [42].

To date, microscopic morphology is regarded as the gold standard for diagnosis [43]. Worldwide, researchers are working on AI-based image analysis for the diagnosis of several oral lesions. Hence, this technology can assist an oral pathologist in making fast decisions regarding patient's histopathology reports and further investigative examinations. AI was introduced in the oral pathology domain to overcome the variability of morphologic diagnosis and to provide consistent and reliable diagnostic reports [44].

Recent research has demonstrated the role of ML in identifying, classifying, diagnosing, and differentiating different oral lesions [45]. A recent study employed CNN for the detection of keratin pearls. The researchers suggested a two-stage method for computing oral histology images. The first stage involved a 12-layered deep CNN for the segmentation of constituent layers. The keratin pearls were diagnosed in the second stage with the help of texture-based feature-trained random forests. The keratin pearls were diagnosed with 96.88% accuracy [46]. Farahani et al. examined the utilization of the Oculus Rift device for the evaluation of digital pathology slides. In their study, all three reviewers established that digital pathology slides were viewed on a VR platform with the Oculus Rift DK2 [47].

Datasets used in AI and oral cancer research are clinical photographic images, patient’s geographic data and habit history, H&E-stained histopathological images, immunostained images, saliva metabolite data, and gene expression data [48]. The limitation of the AI approach is its two-dimensional format. However, the main advantage of the AI approach in image diagnosis is that it overcomes the inconsistency of inter- and intra-observer variability [49].

**Figure 6:**
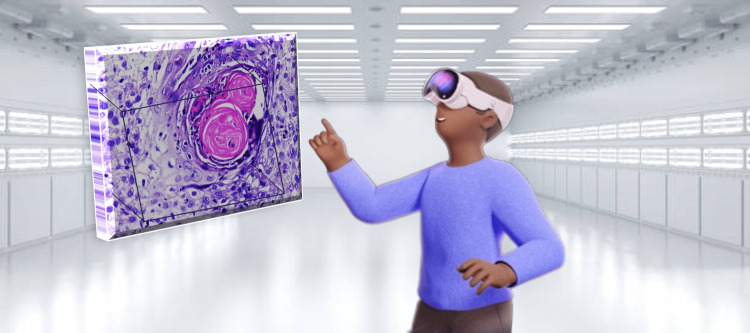
An oral pathologist evaluating histopathological slide incorporated with augmented reality technology. Image Credits: Ishita Singhal and Geetpriya Kaur

The oral pathologist can be taught laboratory techniques such as tissue processing, H&E staining, and special staining techniques with the VR training stimulators. Additionally, the common diagnostic techniques at the microscopic level, such as immunohistochemistry (IHC), fine needle aspiration cytology, and fluorescence in situ hybridization (FISH), can also be duly explained with VR stimulators. The mixed reality systems can also be incorporated into dental schools to explain detailed and labeled histopathological features as well as images of various oral diseases. Moreover, the AR technology can also be used for teaching cytology slides of oral potentially malignant disorders and oral cancer cases to undergraduate and postgraduate students (Figure 6).

Advantages and drawbacks of AI, VR, AR, and MR in oral diagnostics

In the context of a growing global population and the resulting surge in healthcare demands, digital technologies have emerged as indispensable resources for managing the impending influx of patients. Notably, VR and AR platforms offer several advantages across various diagnostic fields. Their applications carry significant potential, particularly in the education of undergraduate and postgraduate students, through the implementation of interactive learning methodologies with clear, objective evaluation criteria. Furthermore, the integration of AI and automation can assume a critical role in safeguarding our healthcare workforce, especially in light of the sacrifices made by many during the COVID-19 crisis.

VR, in particular, holds promise in delivering training to healthcare professionals, covering a spectrum of procedures, from laparoscopic surgery to the evaluation of medical databases. It also plays a pivotal role in formulating treatment strategies and facilitating the rehabilitation of patients dealing with conditions such as autism, cancer, and psychiatric disorders. Moreover, these technologies facilitate advanced learning opportunities for remote students who may lack the means to travel to different cities for specialized studies. They enable the creation of interactive and engaging clinical modules, particularly beneficial in the context of ruling out differential diagnoses in complex medical cases.

However, it is essential to acknowledge that these novel technologies do come with certain limitations when applied in clinical settings. Independent research teams focusing on VR and AR may face challenges when relying on customized augmented systems in intricate experimental models, limiting their widespread applicability. While these technologies can find utility in simpler experimental models, their comprehensive integration into complex scenarios remains a subject of ongoing exploration.

Furthermore, it is worth noting that the current body of research predominantly emphasizes technical skills, particularly in the realm of dentistry, where virtual oral surgery stimulators offer some degree of skill development. Nevertheless, a critical gap exists when it comes to addressing non-technical skills, such as cognitive development, teamwork, interpersonal communication, and emergency management, which are equally vital in clinical practice. To fully harness the potential of VR and AR in health care and dentistry, there is a pressing need for additional research efforts to validate their efficacy and enhance the overall quality of treatment and healthcare delivery through augmented techniques.

## Conclusions

Technological advancements in AI, VR, AR, MR, and XR have revolutionized dentistry, ushering in an era of precision, enhanced patient care, and improved education. These innovations are not here to replace human jobs but rather serve as vital tools in delivering more efficient and cost-effective patient care. These technologies have already transformed various aspects of oral health care, from diagnostics and treatment planning to surgeries and patient experiences, with the potential to eliminate traditional tools like drills and injections. In oral medicine, VR and AR enable 3D-augmented clinical histories, aid in provisional diagnoses, and enhance treatment plan explanations, while haptic-based VR training enhances diagnostic skills. AI, particularly through convolutional neural networks, has improved image interpretation and diagnostic accuracy in oral radiology, leading to precise treatment planning. Oral surgery benefits from AR, VR, and MR in resident training, surgical procedures, and patient education, allowing for greater precision in surgery planning and ensuring patients comprehend expected outcomes. In oral pathology, AI-based image analysis provides consistent diagnostic reports, while VR and AR stimulators assist in teaching laboratory techniques and explaining histopathological features. However, these technologies have limitations, including the need for further validation and addressing ergonomic challenges and high costs. In conclusion, the integration of AI, VR, AR, MR, and XR into dentistry represents a transformative moment, empowering healthcare professionals to deliver superior care and cost savings. Ongoing research should focus on harnessing these tools in oral medicine, radiology, surgery, and pathology to fully unlock their potential in oral health care, promising a brighter, technologically enhanced future for dentistry.
